# Coagulation Profile of COVID-19 Patients

**DOI:** 10.3390/life12101658

**Published:** 2022-10-20

**Authors:** Georgia Kaiafa, Christos Savopoulos, Eleni Karlafti, Konstantina Pantazi, Daniel Paramythiotis, Evanthia Thomaidou, Stylianos Daios, Eleftheria Ztriva, Michalis Gionis, Varvara Fyntanidou, Helena Argiriadou, Triantafyllos Didangelos

**Affiliations:** 1 1st Propaedeutic Department of Internal Medicine, AHEPA University General Hospital, Aristotle University of Thessaloniki, 54621 Thessaloniki, Greece; 2 Emergency Department, AHEPA University General Hospital, Aristotle University of Thessaloniki, 54621 Thessaloniki, Greece; 3 1st Propaedeutic Surgery Department, AHEPA General University Hospital, 54621 Thessaloniki, Greece; 4 Department of Anesthesia and Intensive Care, AHEPA University General Hospital of Thessaloniki, Aristotle University of Thessaloniki, 54621 Thessaloniki, Greece; 5 Vascular Surgery Department, General Hospital of Thessaloniki AHEPA, Aristotle University of Thessaloniki, 54621 Thessaloniki, Greece

**Keywords:** COVID-19, cytokine storm, complement system, thromboinflammation, coagulopathy, COVID-19 biomarkers, microthrombosis

## Abstract

Coronavirus disease is a viral infection that can affect multiple systems and be expressed with many—or no—symptoms. The viral infection begins when the virus binds to the host’s receptor and from that point on, it is transmitted to the rest of the body, where it causes inflammatory reactions. Among other tissues and systems, SARS-CoV-2 impacts the coagulation system, where it triggers the immunothrombotic response. Its effects are rather intense and can lead to many complications. COVID-19-associated coagulopathy is frequently observed in hospitalized patients, especially ICU patients, and can be proven detrimental. It is usually accompanied by other complications, such as sepsis-induced coagulopathy, disseminated intravascular coagulation and venous thromboembolism. Since all these conditions lead to poor prognosis for severely ill patients, thromboprophylaxis and coagulopathy prognosis are just as important as the therapeutic handling of these patients. Since the beginning of the pandemic, many biomarkers have been considered useful when trying to assess the thrombotic risk of hospitalized patients or evaluate the severity of their situation. At the same time, many drugs have already been tested—while others are still being trialed—in order to find the optimal therapy for each urgent situation.

## 1. Introduction

In the last few years humanity has been asked to face COVID-19 and all its consequences. This highly contagious disease was first detected in 2019 in Wuhan of China, when a coronavirus named “severe acute respiratory syndrome coronavirus 2” or “SARS-CoV-2” got linked with cases of pneumonia. Shortly after, at the beginning of 2020, the coronavirus disease spread worldwide and was officially declared as the “COVID-19 pandemic” by the World Health Organization (WHO) on 11 March [1].

COVID-19 has been a significant threat to global health due to the fact that it is not just a respiratory infection, but a systemic disease associated with multiple symptoms in the cardiovascular, gastrointestinal, neurological, immune and hematopoietic system [2]. Even though many COVID-19 patients—mostly young in age—were asymptomatic, many others had a range of symptoms, varying from coughing to diarrhea.

As a matter of fact, COVID-19 is also highly associated with the hematopoietic system and hemostasis [2]. To be more specific, this infection affects blood coagulation by causing endothelium dysfunction and abnormalities of blood cells, and by leading to inflammatory activation of multiple pathways, which are signs of high prothrombotic status. All these alterations are also accompanied by abnormalities in the laboratory exams of COVID-19 patients, such as huge elevation in D-dimers and fibrinogen levels. The clinical outcome is COVID-19-associated coagulopathy and venous thromboembolism (VTE), which are commonly found in hospitalized patients—and especially Intensive Care Unit (ICU) patients [3]—and can have a detrimental outcome. It is important to mention, that similar complications are more likely to occur in patients with pre-existing comorbidities, such as cardiovascular disease [4]. According to Lelapi et al. [4], there is a high correlation between a compromised vascular system and severe COVID-19 infection with complciations [4].

The goal of this review is to analyze the coagulation profile of COVID-19 patients, to describe some SARS-CoV-2 associated clinical situations and to elaborate on the current and future directions for treating critically ill COVID-19 patients.

## 2. Materials and Methods

Before synthesizing this review, comprehensive research on the coagulation profile of COVID-19 patients was performed. All the research was conducted from December 2019 until June 2022, using the PubMed database. All the sources were in the English language. Multiple reviews and original articles were thoroughly studied in order to synthesize a holistic review of the coagulation system reaction during COVID-19 infection.

Since the goal of this review is to exhibit a complete depiction of the coagulation system during SARS-CoV-2 infection and bearing in mind that the COVID-19 pandemic is still active, no limitations were set regarding the number of sources or the date of their release. Moreover, keeping in mind that the COVID-19 pandemic afflicted most countries, no geographical limitations were set either. It is also important to mention that our goal was not only to refer to mild COVID-19 infection, but to emphasize on ICU cases. Other than that, part of our research also focused on the biomarkers taken into consideration during COVID-19 associated coagulopathy, as well as on the possible complications from the coagulation system and the prophylaxis and treatment choices.

The main keywords and key phrases used as a basis for our research were “COVID-19”, “SARS-CoV-2”, “coagulation”, “coagulopathy”, “COVID-19 associated coagulopathy”, “thrombogenesis”, “thromboprophylaxis”, “VTE”, “laboratory exams for COVID-19”, “biomarkers for COVID-19” and “COVID-19 and ICU”.

## 3. Results

To completely understand and explain the pathophysiology behind COVID-19-associated coagulopathy, we first established the effects of SARS-CoV-2 infection on the human body. The virus targets ACE2 receptors and downregulates them, therefore causing deregulation in the RAAS and the KKS pathways [5]. When these pathways are not strictly followed, reactions such as hypertrophy, hyper-inflammation, apoptosis, hypertension and coagulation can occur [5]. During our research, we observed that specific characteristics, such as age, comorbidities, obesity, smoking and ICU hospitalisation, can be considered risk factors for severe COVID-19 infection [6]. At the same time, the ABO blood group, as well as pre-existing liver damage, can also affect the progress of the infection and is related to the chance of coagulopathy development [7].

The thromboinflammation that occurs after the viral infection is followed by several pathways and reactions, which eventually lead to a thrombotic state. The first step after the infection is the activation of innate immune response, through the cytokine storm and complement activation [8]. These situations are part of the immune system, however, when overactivated, they can have detrimental effects, including thrombosis, DIC and cell death [8]. NETs hold a pivotal role in the innate immune response as well, however, they have been proven to be rather cytotoxic and pathogenic [9]. Other than activation of innate immunity, COVID-19 infection has some effects on Virchow’s triad, such as deregulation of the blood flow and its coagulability, as well as inflammation and abnormalities on the endothelium, which is followed by the release of many important biomarkers [10]. Through our research, we found out that some of the most common biomarkers studied during COVID-19 infection include D-dimers, aCL, aPTT, PAI, TAT, VWFAg, VWF, FII, FV, FVIII and PMPs [11,12,13,14,15,16,17,18,19,20]. These biomarkers can give a significant direction on the level of the infection and the condition of the patient. In rather severe cases, the reaction of thromboinflammation can lead to further complications such as DIC, SIC, VTE [21].

When it comes to coagulation treatment and prophylaxis, there are a few possible therapies, which of course depend both on the patient and the risk factors, as well as the level of the infection. Thromboprophylaxis is necessary, especially for ICU patients and should be used in all hospitalised cases [15]. As for the treatment strategies, the use of heparin is a quite common approach [22], while many also suggest iron chelation [8], convalescent plasma therapy [23], antiplatelets [23], anti-inflammatory agents [23], hemostatic modulating agents [23], targeted immunomodulatory therapies [23] and the use of activated protein C [23].

## 4. Discussion

### 4.1. SARS-CoV-2 and COVID-19 Infection

#### 4.1.1. SARS-CoV-2 Infection and ACE2

**Figure 1:**
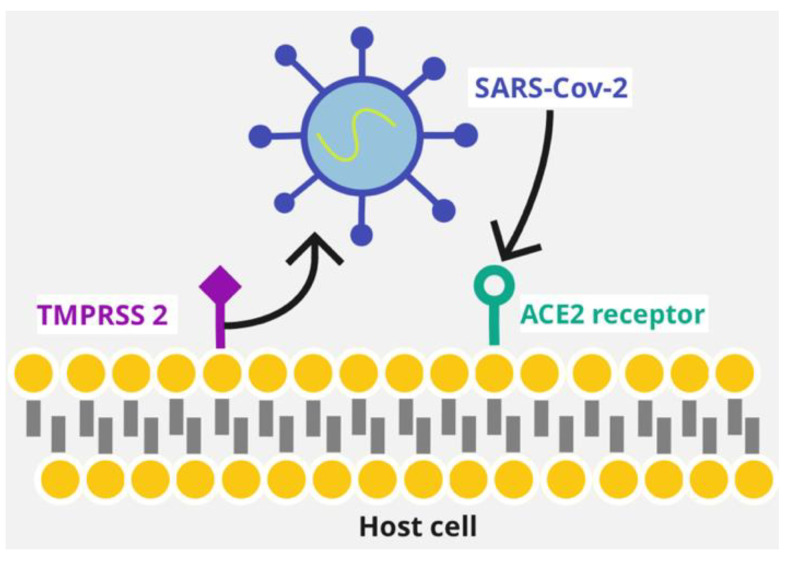
SARS-CoV-2’s binding to the host receptor. SARS-CoV-2 binds to the host cell ACE2 receptor, after TMPRSS2 primes the viral S protein. Abbreviations used: TMPRSS2: transmembrane protease serine-2; ACE2: angiotensin-converting enzyme 2.

The virus entry is initiated when the S1 subunit of the SARS-CoV-2 viral spike (S) glycoprotein binds to the host receptor ACE2 (angiotensin-converting enzyme 2) (Figure 1). As a matter of fact, the S1 protein and receptor interaction is pivotal for determining the SARS-CoV-2 infection [5]. However, it is also important that the S protein is primed by transmembrane protease serine-2 (TMPRSS2) before it binds to the ACE2 receptor and enters the host cell [24] (Figure 1).

**Figure 2:**
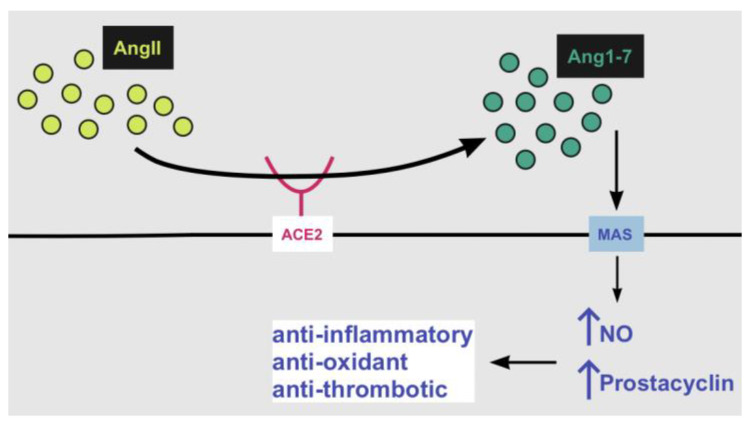
ACE2 converts AngII to Ang1-7. Ang1-7 then binds to MAS, causes an elevation of NO and prostacyclin and thus has anti-inflammatory, antioxidant and anti-thrombotic effects. When SARS-CoV-2 binds to the ACE2 receptor, this pathway is downregulated. Abbreviations used: MAS: a transmembrane G-protein coupled receptor; AngII: angiotensin II; Ang1-7: angiotensins 1-7; NO: nitric oxide.

In general, ACE2 regulates blood pressure and inflammation and in the absence of SARS-CoV-2, ACE2 stops angiotensin II (AngII) and its proinflammatory activity. To be more specific, this enzyme binds to AngII and converts it into the anti-inflammatory peptide angiotensin 1-7 [25] (Figure 2). In contrast, when ACE2 is attached by the SARS-CoV-2 S protein, its functionality alters [25].

After the infection, the ACE2 downregulation caused by SARS-CoV-2 leads to the deregulation of both the RAAS (Renin-Angiotensin-Aldosterone-System) and the KKS (Kinin-Kallikrein system) pathways, which can result in severe illness and a fatal outcome [5].

#### 4.1.2. ACE2 and RAAS Pathway

When it comes to the RAAS pathway, as already mentioned, ACE2 catalyzes AngII’s conversion to Ang1-7. Ang1-7 binds and activates a transmembrane G-protein coupled receptor (GPCR) named MAS [26] (Figure 2). This binding results in an increased production of nitric oxide (NO) and prostacyclin and thus a restriction of platelet activation. In other words, Ang1-7’s reaction with MAS has anti-inflammatory, antioxidant and anti-thrombotic effects, which are pivotal [5,26] (Figure 2). Other than that, ACE2 also mediates the conversion of AngII to Ang1-9, which then can be converted to Ang1-7 by ACE [5]. Through this mechanism, ACE2 prevents the possible effects of AngII and has a counterbalance role in the RAAS pathway [5].

**Figure 3:**
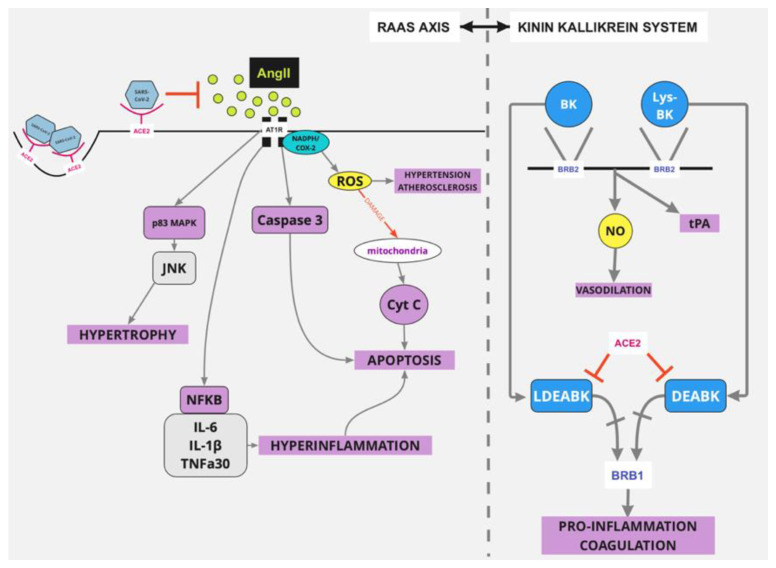
SARS-CoV-2 infection and alterations in the RAAS and KKS axis. SARS-CoV-2 binds to the ACE2 receptor causing its internalization. ACE2 no longer converts AngII to Ang1-7 and AngII binds to the AT1 receptor and subsequently triggers the production of p83 MAPK, caspase 3, ROS and Cyt C, which promotes apoptosis, hyperinflammation and hypertrophy. ACE2 also affects the KKS axis by affecting DEABK’s and LDEABK’s attachment to BRB1, thus leading to inflammation and coagulation. Abbreviations used: RAAS: Renin-Angiotensin-Aldosterone-System; AT1R: Angiotensin type 1 receptor; p83 MAPK: p83 mitogen activated protein kinase; Cyt C: Cytochrome C; ROS: Reactive oxygen species; KKS: Kinin-Kallikrein system; DEABK: [des-Arg9]-BK; LDEABK: Lys-[des-Arg9]-BK; NADPH: nicotinamide adenine dinucleotide phosphate; COX2: cyclooxygenase 2; IL-6, IL-1β, TNFa30: proinflammatory cytokines; BRB1/2: bradykinin receptor B1/B2; BK: bradykinin; Lys-BK: Lys- bradykinin.

The RAAS pathway is mainly responsible for blood volume regulation and systemic vascular resistance. It also plays an important role in sodium reabsorption, inflammation and fibrosis [5]. This pathway is affected by COVID-19 through the following sequence: When SARS-CoV-2 binds to ACE2, there is an accumulation of AngII, a decrease of Ang1-7 and an unperturbed RAAS activation via the AngII/angiotensin type 1 receptor (AT1R) (Figure 3). This can lead to vasoconstriction, oxidative stress, inflammation, atrophy and fibrosis [5].

Other than that, AngII can cause endothelial dysfunction by activating cyclooxygenase-2 (COX-2), which subsequently creates vasoactive prostaglandins and reactive oxygen species (ROS), mediated by NADPH oxidases [5,26] (Figure 3). This excessive activation of the AngII/AT1R/NADPH (nicotinamide adenine dinucleotide phosphate) chain has been associated with hypertension and atherosclerosis [5]. The overproduction of ROS and the simultaneous deficiency of NO can lead to severe SARS-CoV-2 infection and have detrimental effects on the endothelium [26]. This axis also induces the release of cytochrome C from damaged mitochondria and subsequent apoptosis (Figure 3). Additionally, AngII’s biding to AT1R also triggers the activation of caspase 3—which leads to apoptosis—as well as the activation of p38 MAPK (mitogen activated protein kinase) and JNK (Jun N-terminal kinase) cascade—which can cause hypertrophy [5] (Figure 3).

Moreover, AngII can also trigger NF (nuclear factor) kappa b’s activation, which is responsible for the transcription of proinflammatory cytokines, such as IL-6, IL-1β and TNFa30 [27], which—in addition with AngII’s other effects—cause the hyperinflammation that is observed in the late phase of COVID-19 infected patients [5] (Figure 3).

Understanding ACE2’s important role in pathophysiology of all the above-mentioned pathways was very valuable, mainly at the beginning of COVID-19, when there were research efforts to provide soluble recombinant (r)ACE2 in order to address both mechanisms by cell independent binding at SARS-CoV-2 and degrading AngII to Ang1-7 [28].

#### 4.1.3. ACE2 and KKS

The KKS is very important for regulating several processes, such as coagulation, inflammation and pain [5]. Its main role is to produce peptides, like bradykinin (BK) and LYS-BK, which attach to the bradykinin receptor B2 (BRB2) [29] and increase nitric oxide (NO) production (Figure 3). NO acts as a vasodilator, which counteracts RAAS’s effect as a vasopressor [5]. Other than that, BK also induces an increase in tissue plasminogen secretion (tPA) and thus controls thrombogenesis (Figure 3). The KKS also regulates the production of [des-Arg9]-BK (DEABK) and Lys-[des-Arg9]-BK (LDEABK) [29], which act as proinflammatory factors (by inducing the release of cytokines) by binding to the bradykinin receptor-B1 (BRB1) [5] (Figure 3).

ACE2 does not interact with BK and Lys-BK, but it affects the DEABK/LDEABK/BRB1 pathway by cleaving the residue in DEABK and LDEABK and affecting their attachment to the receptor. Therefore ACE2’s interaction with SARS-CoV-2 results in an alteration in the KKS, which is the overactivation of the DEABK/LDEABK/BRB1 axis and the subsequent boost in inflammation [30] and coagulation [5] (Figure 3).

#### 4.1.4. COVID-19 and Coagulopathy Risk Factors

While most COVID-19 patients have mild symptoms, there are some cases when COVID-19 can lead to severe pneumonia or death. The main risk factors for such cases include 60 or more years of age, smoking, obesity and pre-existing comorbidities [6]. Thrombosis related COVID-19 risk factors are immobilization, systemic inflammatory status, mechanical ventilation, ICU hospitalization, central vein catheters installation, and an increase in certain biomarkers [12,31].

A special reference should be made to hypoxia, as it may trigger thrombogenesis after COVID-19 infection [32]. Hypoxia mainly occurs in moderate or severe COVID-19 cases and can result in endothelial malfunctions and hypercoagulability [33]. COVID-19 patients with hypoxia appear to be suffering from prothrombotic conditions, through the upregulation of PAI-1 and the stimulation of procoagulants synthesis, like TF and von Willebrand Factor (vWF) [34].

Another study focused on the correlation between COVID-19 and ABO blood group [35]. This trial by Zhao et al. [7], which took into consideration 2173 patients, showed that group A patients were more likely to be symptomatic COVID-19 patients than group O patients. Similarly, a European study confirmed the higher risk of group A individuals—in comparison to other blood groups—and a higher protective effect in blood group O patients [36]. In general, ABO is a pleiotropic locus that is related to thrombotic diseases, such as VTE [33]. It is believed that the difference in thrombotic risk levels is associated with VWF levels—which are lower in group O patients—and IL-6 levels [37].

One last risk factor for thrombosis during COVID-19 infection that is worth mentioning is liver damage [10]. Multiple studies have shown that the infection of the liver by the virus includes elevation of alanine aminotransferase, aspartate aminotransferase and bilirubin [10]. Since the liver is the main source of hemostasis and plasma proteins, an affected liver can lead to rather harmful dysregulations of the coagulation system [10].

### 4.2. COVID-19 and Thromboinflammation

When it comes to the hematopoietic system, COVID-19 can be considered a prothrombotic disease. It is crucial to explain the events, which lead to thrombogenesis after COVID-19 infection, especially when it comes to patients in the ICU. The effects of the SARS-CoV-2 infection on the human organism begin during the early phases of the disease and the incubation period. During that time, symptoms are absent and peripheral blood leukocyte and lymphocyte counts are mostly normal [2]. Shortly after viremia occurs and the virus spreads, especially to the tissues that express high levels of ACE2. Later, 7 to 14 days after the first symptoms appear, an increase in systemic inflammatory mediators takes place and significant lymphopenia occurs, which is often associated with the need for ICU care [2].

#### 4.2.1. COVID-19 Infection and Innate Immune Response

##### Cytokine Storm

**Figure 4:**
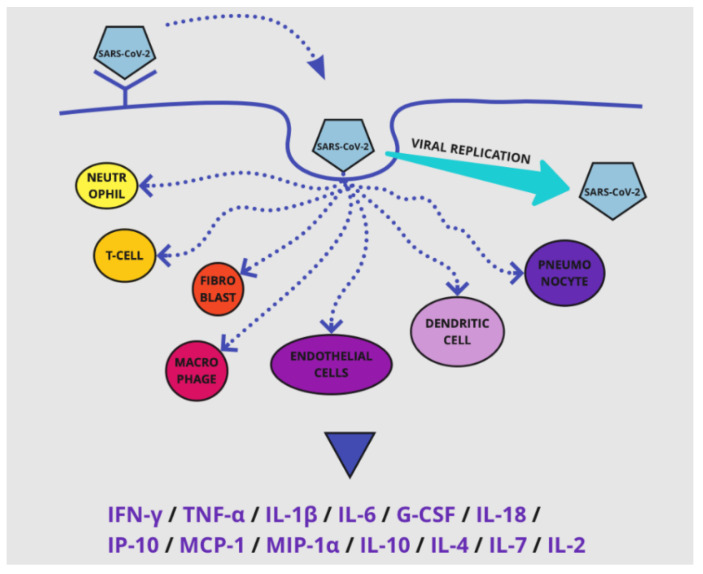
The cytokine storm. Viral entry in the cell after binding to the ACE2 receptor. After entry, the virus replicates inside the cell and activation of host innate immunity occurs. This contains the release of pro-inflammatory cytokines, which when uncontrolled leads to the cytokine storm. *Abbreviations used*: IL: interleukin; IP: interferon gamma-induced protein; MCP-1: monocyte chemoattractant protein 1; MIP: macrophage inflammatory protein; IFN-γ: interferon gamma, TNF-α: tumor necrosis factor alpha.

In COVID-19, thromboinflammation and coagulation malfunction emerge through specific pathways, like the one of inflammation and the cytokine storm [14]. The SARS-CoV-2-induced activation of innate immunity results in a rise of pro-inflammatory cytokines production—by cells like neutrophils, T-cells, macrophages, fibroblasts, pneumonocytes, endothelial cells and dendritic cells [14] (Figure 4). It is essential to emphasize that even though cytokine release plays an important role in the immune system and is subsequently desirable, its overactivation can do severe damage to many systems [15].

Specifically, in COVID-19, the pro-inflammatory cytokines that are notably overexpressed as part of the cytokine storm appear in Figure 4. It is worth mentioning that out of all the cytokines mentioned, IL-6 and IL-2 are strongly associated with the severity of the disease, meaning that they are more elevated in critically ill COVID-19 patients than in ordinary COVID-19 patients [15]. The explosive release of these pro-inflammatory cytokines provokes a major expression of the biological parameters of inflammation [3]. Meanwhile, studies have shown that in vivo, higher levels of specific factors, such as TNF-a, IL-6 and IL-1, can be found in patients with inflammatory conditions (ex. sepsis) in addition to a hypercoagulable status [38]. IL-6 has many roles in the inflammatory process. For instance, it induces the production of TF in macrophages during inflammation [12]. In the plasma of COVID-19 patients, the infection-related elevation of IL-6 and IL-6R results in a boost of endothelial cells and TF [14] and this infection-induced coagulopathy may play a pivotal role in thrombocytopenia, while the cytokine storm triggers the thrombocytosis [14]. Other than that, IL-6 also partakes in the synthesis of other coagulation factors, such as fibrinogen and factor VIII (FVIII) [14,39], and promotes the secretion of VEGF (vascular endothelial growth factor), when acting on endothelial cells, thus inducing vascular permeability [14].

Besides this, IL-6, IL-1α is vastly expressed by activated platelets, endothelial cells and monocytes during the pro-inflammatory phase and associates the coagulation system with the inflammation [15]. IL-1α is present in thrombo-inflammatory conditions like sepsis, where it is released by epithelial cells and has the role of an alarmin; it activates the inflammatory cascade [15]. Meanwhile, when expressed by endothelial cells, IL-1α affects thrombogenesis by recruiting granulocytes, increasing the clot-lysis time and the platelet activity [15]. One could say that IL-1—accompanied by TNF—is one of the main mediators that suppress the endogenous coagulation cascade [12].

##### Complement Activation

The complement system is a crucial part of the innate immune response during COVID-19 infection [26]. Data suggest that the complement system is also involved in the pathogenesis of thrombosis in COVID-19 patients, in disorders like endothelial damage, thrombosis, thrombocytopenia and hemorrhage, which are mortality risk factors for critically ill COVID-19 patients [8].

The complement system shares three pathways, through which it activates plasma proteins for host defense. The first pathway is triggered by host-antigen contact, the second one is activated by antigen-antibody complexes, while the third one is the lectin pathway, which binds polysaccharides on antigen surfaces to host cells [8]. In the third pathway, the virus invades and damages host cells that express the ACE2 receptor, thus causing a thromboinflammatory response, which further activates the complement system [40]. In all 3 of the pathways mentioned above, C3 and its derivatives are the main elements of the complement system [8]. What is rather intriguing, is the fact that in a study executed on mice infected with SARS-CoV-1, it was proven that mice lacking the complement C3 factor underwent less severe respiratory inflammation [41]. Even though similar clinical trials have yet to be run on mice with SARS-CoV-2, the study mentioned above suggests that excessive complement activation plays a significant part in the dysregulation of the immune response [26].

In COVID-19, the complement system has a procoagulant effect through the action of mannose-associated serine protease-2 (MASP-2), which is a component of the lectin pathway, and its role is to activate thrombin and to form a fibrin mesh [8]. Other than that, complement action can foster endothelium changes, which affect the proteins involved in the clotting cascade. Complements further promote coagulation by initiating platelet accumulation. Another path, through which the complement system can lead to thrombogenesis, is by getting activated by the coagulation axis. Specifically, the activation of factor XIIa (FXIIa), which occurs in the coagulation axis, can subsequently activate the complement complex C1 [42]. Therefore, it is obvious that the complement and coagulation pathways are closely interlinked [8].

At the terminal phases of complement activation C5b-9, C3b and C5b fragments are released [8]. These terminal subunits aid in the synthesis of prostaglandins and leukotrienes, which, afterward, induce the production of pro-inflammatory cytokines, to induce inflammation and protect the host from the viral infection [8]. When this complement-induced cascade of immunity is not controlled, it can incite inflammation, disseminated intravascular coagulation (DIC) and cell death. This demonstrates that the complement system, while important for immunity, can also turn out to be detrimental through its disease-inducing pathways [8]. In order to prevent conditions such as such, many suggest the use of anticomplement agents early on, to limit cell and tissue damage.

##### NETs

Alongside complement’s action, the neutrophils, which produce TF and release neutrophil extracellular traps (NETs) that carry active TF, are also a part of the innate immune response, which results in a hyperinflammatory reaction and thrombotic microangiopathy [9,13,15]. NETs—besides their main role—are also capable of initiating inflammation and thrombosis [9]. Specifically, NETs transfer multiple oxidant enzymes such as NADPH oxidase and nitric oxidant synthase to the extracellular space, while also holding their role as a source of extracellular histones with significant cytotoxic potential [9].

They can cause cardiovascular disease by spreading inflammation in vessel walls, while they can also cause blockage in veins, arteries and microscopic vessels when formed intravascularly [9]. As a matter of fact, NETs are present in thrombi and enhance prothrombotic pathways. NETs can also activate the generation of thrombin, but only when they stand as individual NET components of cell-free histones and DNA [15].

NETs partake in pathologies like sepsis and thrombosis when they are produced excessively, a condition named NETosis [26]. What exactly happens is that NETs themselves contain prothrombotic molecules, such as factor XII, vWF, TF and fibrinogen, which trigger thrombogenesis. Simultaneously, circulating histones which are NETs’ components, trigger Toll-like receptors on platelets and result in thrombus [26]. In general, it is highly believed that NETosis is a risk factor when it comes to thrombosis in COVID-19. This is also the reason why many therapeutic strategies aim to combat NETs or take advantage of NET inhibition [9,43]. Some approaches aim to prevent the release of NETs with neutrophil elastase inhibitors and adenosine receptor agonists. Special attention should be given to patients with hyperactive neutrophils since they might be at high risk of thrombotic events and need a more aggressive anticoagulant treatment when hospitalized [9].

### 4.3. COVID-19 and Virchow’s Triad

#### 4.3.1. Abnormalities on the Endothelium/Vessel Wall Abnormalities

As a matter of fact, endothelial dysfunction might trigger systemic thrombotic events [44]. In general, endothelial dysfunction leads to high levels of D-dimers, thrombin and fibrin degradation products, thrombocytopenia and prolonged clotting times, which then result in thrombosis and vascular occlusive events and finally hypoxia and pulmonary congestion [12]. Research has shown that SARS-CoV-2 can affect endothelial cells both in vitro [45] and in patients infected with COVID-19. The replication of the virus within the endothelial cells is what causes the procoagulant reaction [46].

SARS-CoV-2 can affect the endothelium through a series of afore mentioned inflammatory pathways triggered by immune response [13]. More specifically, innate inflammation-induced cytokines activate endothelial cells and endothelial injury [16]. The local inflammation impels vascular permeability and at the same time, it impairs the production of NO and prostacyclin I, which have antithrombotic properties [19]. These pathways trigger multiple downstreams, which all lead to coagulopathy. These induce dysregulation of the endothelium, leukocyte activation, neutrophil extracellular traps (NET) and tumor necrosis factor-a (TNF-a) [47] production, enhanced PAI-1 (plasminogen activator inhibitor-1) expression [19], vWF release [19], complement deposition and platelet activation [13]. Meanwhile, thrombodulin’s activity and t-PA (tissue plasminogen factor) decrease [19]. Generally, when a vascular endothelial injury occurs, it causes further thrombocytopenia and a decrease in natural anticoagulants, as well as a hemostatic activation [16].

The endothelium is coated by glycocalyx, which releases tPA (tissue plasminogen activator), and thus provides an antithrombotic surface to the endothelium and regulates vascular blood flow. The glycocalyx is rather fragile and therefore is easily corrupted by COVID-19 [48], which causes injury of cellular membranes and thus various clotting cascades [26]. Moreover, since it’s so fragile, its components are used as biomarkers of endothelial damage in various infections [10]. In other words, endothelial dysfunction and damage is followed by endothelial expression of prothrombotic molecules and receptors, such as P-selectins, angiopoietin-2 and endothelin-1, which actively contribute to thrombosis [26]. Other than these molecules, the endothelial disruption causes a massive release of the procoagulant factor VIII, along with vWF from Weibel-Palade bodies [10,26].

When it comes to vWF, it seems to be associated with coagulation mechanisms in COVID-19 infection. After endothelial dysregulation occurs, there is a boost in the release of subendothelial vWF, which is multimerized and activated. Upon activation, vWF exposes both its platelet-binding and collagen-binding domains and uses them as molecular glues that attach platelets and subendothelial collagens together [26]. As a result, platelet aggregations and thrombosis take place [26]. It is rather interesting to mention that a recent study led to the conclusion that vWF antigen and activity were three times higher in non-intensive care unit (ICU) COVID-19 patients, compared to both the control group and ICU patients [26]. This suggests that both critically and non-critically ill patients with COVID-19 can have a high thrombotic risk [26].

#### 4.3.2. Abnormal Blood Flow

COVID-19 is linked to hyper-viscosity, which predisposes one to thrombosis and causes endothelial injury [10]. The elevation in blood viscosity is justified by the high levels of fibrinogen that are observed in COVID-19. In multivariate Cox analysis, the fibrinogen-to-albumin ratio (FAR) is a predictor for disease progression [49]. Dysregulation of blood flow can also be observed in the microcirculation, through the formation of microthrombi and angiogenesis that ruin microcirculation in COVID-19 [10].

A special reference to ICU patients should be made since the abnormal blood flow during COVID-19 hospitalization can be attributed to the immobilization and the angiopathies that are a result of the exacerbation of chronic systematic diseases—such as heart failure and diabetes mellitus—and age-related fragility [19].

#### 4.3.3. Blood Hypercoagulability

Blood hypercoagulability can be an outcome of inhibition of the plasminogen system. Upon SARS-CoV-2’s binding to the ACE2 receptor and the receptor’s internalization, undisturbed AngII action occurs through the AT1 receptor, which leads to thrombus formation. This happens because ACE2 tends to activate tPA—which prevents thrombi –, while AngII and AT1R trigger the release of PAI-1 [10]. This promotes coagulation. One could say that the ACE2’s takeover by SARS-CoV-2 alters the tPA/PAI-1 balance and provokes a prothrombotic state [10].

Moreover, the hypercoagulable state can also be accredited to platelet dysfunction. The platelet-to-lymphocyte ratio (PLR) is used as a marker for inflammation and can help predict thrombosis [10]. When referring to COVID-19, PLR is elevated, and platelet activation occurs through the activation of AT-1R and its release of PAI-1. Furthermore, in COVID-19, platelets are also triggered by the altered ACE2R function [10]. Platelet activation is so important in COVID-19 coagulopathy because platelets have Mas receptors that modify thrombosis via the release of NO and thus contribute to clots [10]. A meta-analysis of 7613 COVID-19 patients revealed that severely ill COVID-19 patients had lower platelet count than patients with non-sever infection, while the COVID-19 non-survivors had an even lower platelet count [50,51]. In a parallel manner, patients with severe pneumonia—but not COVID-19—had fewer platelets than COVID-19 patients, a find that proves that platelet activation is indeed present in COVID-19 [52].

Other than that, blood hypercoagulability and the subsequent prothrombotic state are also a result of complement activation and systemic immune reactions, while some studies support that antiphospholipid syndrome is also responsible, however, this remains uncertain [10].

### 4.4. The Role of Inflammasomes in the Genesis of Coagulopathy in COVID-19

In general, inflammasomes are multimeric platforms that may form a complex and lead to the production of active IL-18 and IL-1β, when activated by stimuli such as pathogen-associated molecular pattern stimuli (PAMPs) or damage-associated molecular patterns (DAMPs) [53]. Through the stimulation, inflammasomes partake in inflammatory and antimicrobial actions [53]. At the same time, the activation of inflammasomes also triggers, through a number of mechanisms, pyroptosis and thrombosis [53]. As a matter of fact, some inflammasomes such as NLRP3 (NOD-like receptor protein 3), have been described as a signaling connection between inflammation and thrombosis, by adjusting clot shrinkage and platelet activation [54]. Specifically, thrombin binds to a G-protein coupled receptor and leads to ROS-related activation of inflammasomes, which stimulate the release of IL-1β in the cell [54]. IL-1β subsequently triggers the release of IL-6 and therefore of TF, which transforms prothrombin to thrombin [54]. The release of the microvesicles which embody TF is activated by inflammasome-induced pyroptosis [54]. Thrombin holds a pivotal role in fibrinogen conversion to fibrin, thus causing clot formation and coagulopathy [54].

### 4.5. Ischemic Stroke and COVID-19

The incidence of ischemic stroke among COVID-19 hospitalized patients varies from 0.1–6.9%. Corresponding to an overall 1.4%, severe clinical illness and ICU admission seems to be related to a five-fold increase in the risk of ischemic stroke. Among all possible mechanisms, 4 seem to be the main related to thromboembolism and stroke in patients, diagnosed with COVID-19: Immune-mediated thrombosis and hypercoagulopathy, the alternative RAS pathway, cardioembolism and COVID-19 associated cardiopathy and SARS-CoV-2 mediated damage of the neurovascular unit [55].

Some studies suggest that the presence of SARS-CoV-2 in neuronal cells in ischemic areas, proposing a relationship between brain ischemia and SARS-CoV-2 invasion. There is a possibility that CNS involvement in COVID-19 might be secondary to microvascular thrombosis and injury to the neurovascular unit, resulting in blood-brain barrier disruption and viral translocation. The overproduction of proinflammatory proteins and adhesion molecules with the circulation-activated leucocytes, results in the augmentation of the inflammatory process in the ischemic brain and leads to ischemic core expansion [56].

With regard outcomes, according to Ntaios et al. [57], COVID-19-associated ischemic strokes are more severe with more functional outcomes and higher mortality than non-COVID-19 ischemic strokes [57].

### 4.6. Biomarkers/Laboratory Abnormalities in COVID-19 Associated Coagulopathy

In COVID-19 patients, it is very important to run laboratory tests in order to be able to categorize the phase of the infection, to check the progress of the disease and to decide on the best care option at any moment [13]. When a COVID-19 patient is admitted to a hospital, laboratory tests are executed to evaluate the patient’s condition. Each medical team decides on a different combination of tests on an individualized procedure, however, some of the following biomarkers should be checked: anticardiolipin antibody (aCL), anti-b2glycoprotein I (ab2GPI), D-dimers, PT (prothrombin time), aPTT (activated partial thromboplastin time), platelet count, fibrinogen, LA1/LA2/LA3 (lupus anticoagulant 1/2/3), Pr S (protein S), Pr C (protein C), ATIII (antithrombin III), PAI (plasminogen activator inhibitor), TAT (thrombin-antithrombin complex), PIC (plasmin inhibitor complex), VWFAg (von Willebrand factor antigen), VWF (von Willebrand factor), FII/FV/FX/FVII/FVIII/FIX/FXI/FXII (factors) and PMPs (platelet microparticles) [11,12,13,14,15,16,17,18,19,20].

Fluctuations in the biomarkers mentioned above usually appear 7 to 11 days after the first symptoms occur or 4 to 10 after hospitalization begins [12]. Revaluation of patients’ clinical state should be done regularly, and the laboratories should be rechecked every 2–3 days, especially in high-risk COVID-19 patients [12]. It is suggested that in the case of complications, the biomarkers should be checked again after the episode to be able to compare the levels and act accordingly by a multidisciplinary team of specialties. In general, it is uncertain what the most adequate time between tests is and it should be specified according to every patient and based on clinical indications and laboratory possibilities [20].

#### 4.6.1. Test Methods

The tests used are a conventional coagulation test (CC), a platelet function test, a fibrinolysis and endogenous anticoagulation system test, as well as a rational thromboelastometry test or ROTEM [17] and thromboelastography test or TEG [19]. The last two tests are known as global tests of hemostasis (GTH). With CC tests one can easily analyze platelet count, plasma fibrinogen concentration, ILs, aPTT, PT, TT (thrombin time) and INR [17]. However, it is important to mention that markers like the ones measured with CC tests are not enough to reveal the level and complexity of hemostasis in ICU patients [17]. For an assessment as such, a ROTEM test is needed. ROTEM tests give a representation of the viscoelastic properties of the patients’ blood and evaluate clot progression times [58]. A study done by Pluta et al. [19] on ICU COVID-19 patients, led to the conclusion that ROTEM tests can identify elevated coagulation in more than 50% of patients and thus be used to rapidly recognize patients with coagulopathy that are at risk of complications in the coagulation system [19]. ROTEM test alongside with TEG test are methods for quick assessment of the dynamics of clots in COVID-19 patients [19]. Furthermore, GTH—in contrast to CC tests—enables the possibility to diagnose hypercoagulation and fibrinolysis [19].

#### 4.6.2. Fibrinogen and D-dimers

Elevated fibrinogen and D-dimers are two of the most important markers when it comes to hypercoagulability and hemostasis [22]. D-dimers are produced in the blood after the fibrin polymer’s breakdown by plasmin. Fibrin is the molecule that is deposited in ARDS [33]. D-dimers indicate the level of lung injury [11]. In general D-dimers monitor the severity of the inflammation by increasing as the disease progresses (D-dimers > 1.5 μg/L) [11,31]. A progressive increase in D-dimers levels has indeed been observed in COVID-19 patients that require ICU care and not so much in the rest of the patients with COVID-19 [8]. Specifically, D-dimers levels gradually elevate from 1.6 μg/mL to 4.7 μg/mL and to 6.9 μg/mL [8]. Patients with such high D-dimers levels should be—if not already—hospitalized, even in the absence of other symptoms [22]. Cases of extreme D-dimers elevation (>21 μg/mL) have been associated with mortality [59]. D-dimers are a non-specific acute phase reactant and thus changes in their levels can be associated with other inflammations. However, it is believed that a constant increase in D-dimers levels points to a higher risk for thrombosis and coagulation complications during COVID-19 [31]. Some even support that D-dimers levels are a marker for viral load [8].

When it comes to fibrinogen, it is important to check its concentration in COVID-19 patients [22]. Fibrinogen is an acute-phase protein and thus its levels are elevated during inflammation. On top of that, high fibrinogen levels are associated with thrombosis, irrelevantly of the presence of inflammation [22]. High fibrinogen concentrations partake in blood viscosity as well, which as mentioned earlier, is part of Virchow’s triad and can contribute to thrombogenesis [22]. In contrast to D-dimers levels which stay high, studies have shown that fibrinogen levels significantly drop shortly before the death of COVID-19 patients with complications. For this reason, fibrinogen is a great prognostic marker for COVID-19 progression in hospitalized patients [22].

#### 4.6.3. Von Willebrand Factor

Von Willebrand factor is an acute phase reactant and is expected to be elevated during inflammation. It is a glycoprotein produced by endothelial cells and platelets. The FVIII/VWFAg ratio is used to showcase the disease severity [22]. It should be mentioned that VWF can be measured with the vWFAg test.

Von Willebrand factor and ADAMTS-13, which is its cleaving protease, hold a pivotal role in hemostasis [34]. ADAMTS-13 deficiency results in vWF multimers built up and subsequent microvascular thrombosis and thrombocytopenia [34], a condition also known as thrombotic thrombocytopenic purpura (TTP). VWF-induced thrombogenesis can also occur from the elevation of vWF release from Weibel-Palade bodies [34]. This is a result of inflammation, which blocks vWF cleavage by ADAMTS-13. The factors that prevent ADAMTS-13 action are—among others—high concentration of IL-6, the binding of specific molecules on vWF cleavage site of ADAMTS-13 and proteolytic decomposition of ADAMTS-13 by molecules that are elevated during inflammation [34]. In both cases, thrombi arise when vWF interacts with NETs released from neutrophils and creates a base on which platelets can attach [34].

This imbalanced vWF/ADAMTS-13 ratio is often observed in conditions like arterial thrombosis, ischemic stroke, pediatric stroke and even myocardial infraction in young women [34]. Also, a study done by Ma et al. [60] demonstrated that smoking—which is already a risk factor for COVID-19 complications—can induce a decrease in plasma ADAMTS-13 levels [60].

#### 4.6.4. Prothrombin Time, Activated Partial Thromboplastin Time and Platelet Count

Even though in COVID-19 patients there are alterations in D-dimers, fibrinogen and vWF, the levels of PT and aPTT stay relatively normal [22]. As for PT, even in ICU patients and non-survivors, it was only slightly prolonged [22]. Similarly, mildly prolonged was activated partial thromboplastin time [22]. Prolongation of aPTT is likely to be observed, but it is usually a result of the patients’ underlying conditions which produce molecules that elevate aPTT, such as heparin, lupus anticoagulant (LA) and CRP [61]. Shorter aPTT can also be measured and is usually due to the overproduction of VIII factor and fibrinogen in acute inflammation [62]. What is rather interesting is that a study of 184 COVID-19 ICU patients, proved that a major prolongation of PT or aPTT (PT > 3 s and aPTT > 5 s) is indeed a predictor of thrombotic incidents in COVID-19 [13].

Platelet count is another factor that is usually taken into consideration in COVID-19 patients, however, it is of minor importance because thrombocytopenia rarely occurs alongside COVID-19 [22]. A reference to PMPs (platelet microparticles) should also be made. Their coagulation abilities are similar to those of platelets, with the main difference being that PMPs act as procoagulants with a higher activity, which is corresponding to their smaller size [63].

#### 4.6.5. Protein C, Protein S and Antithrombin

Deficiency of certain biomarkers, such as protein C (pr C), protein S (pr S) and antithrombin (AT) can be characteristic of thrombophilia or result in acquired thrombophilia [22]. That is because these biomarkers are used by the coagulation system as natural anticoagulants [22]. Acquired thrombophilia mainly occurs after viral infections, with a prominent one being the SARS-CoV-2 infection [22]. Some studies have related pr S deficiency with severe COVID-19 infection [64], while others have identified decreased AT concentration in COVID-19 non-survivors [65]. Moreover, another study observed high levels of pr C in hospitalized patients and a correlation between pr C concentration levels and the COVID-19 progress, with ICU patients having the highest levels of pr C [66].

#### 4.6.6. Antiphospholipid Antibodies

Antiphospholipid antibodies (aPLs) are present in antiphospholipid syndrome (APS), venous and arterial thrombosis and even microthrombosis [47]. When conducting tests for LA, there are some disadvantages, with the most important being the interference of CRP and anticoagulation therapy, which are both present in COVID-19 patients [22]. Especially CPR, is inevitably elevated in severely ill patients, because of the inflammation. In a study done by Katrien M. J. Devreese, where they tested all three aPLs in COVID-19 patients, there was no obvious association between thrombosis and aPL levels [22]. However, in other studies, there seems to be a belief of association between aPL and thrombogenesis [22]. All in all, even though the coagulation malfunctions that occur in COVID-19 are dependent on many aspects, the involvement of aPL is not quite clear [22]. Then again, in a study by Taha et al. [67], aPLs were detected in almost half of the sample COVID-19 patients, especially in the critically ill, but still, there was no direct association between aPLs and thrombotic complications [67].

### 4.7. COVID-19 Associated Coagulopathy and Complications

Evidence shows that most hospitalized critically ill COVID-19 patients have complications that appear as coagulation disorders—thrombotic or hemorrhagic [8]. As a matter of fact, many COVID-19 non-survivors seem to have had severe coagulopathy, sepsis and ARDS. It is notable that severe illness occurs in 16% of COVID-19 cases, according to Guan et al. [68]. Furthermore, the overproduction of inflammatory cytokines in COVID-19-associated coagulopathy (CAC) can lead to hemophagocytic lymphohistiocytosis (HLH)/macrophage activation syndrome (MAS), which further triggers the coagulation [19]. Thrombotic microangiopathy and APS—which resemble CAC but are not the same—can also occur [19]. Generally, in severe COVID-19 cases, it is likely that hypercoagulability and high D-dimers levels will occur. These complications can lead to major inflammation, pneumonia, progression to ARDS and even death [3]. Risk assessment is necessary.

#### 4.7.1. DIC/Sepsis Induced Coagulation (SIC)

According to the International Society of Thrombosis and Hemostasis (ISTH), DIC symptoms are more common in non-survivors rather than survivors of COVID-19, thus implying a correlation between DIC and COVID-19 mortality [69]. Studies have shown that up to 70% of COVID-19 ICU patients present symptoms and signs of DIC [70]. DIC occurs after activation of the coagulation system and can potentially lead to thrombotic manifestations and bleeding complications [21].

SIC refers to sepsis-induced coagulopathy and is similar to DIC [12]. DIC and SIC presence during COVID-19 is a result of various pathologic mechanisms. Their main characteristics are platelet activation and fibrin deposition [21] as a result of inflammation. The main inflammatory molecules that are active are TNF-α, IL-1 and IL-6, as well as NETs, which partake in the thrombogenesis aspect [21] by inducing thrombocytosis and hyperfibrinogenemia [47]. However, in DIC, thrombin production is mainly triggered by FVII’s activation and the subsequent release of FXa and FIXa [21].

Generally, in DIC, a defect of the natural anticoagulation pathways is common, such as a lack of TF pathway inhibitor, when increased TF-dependent coagulation occurs [21]. Thrombin inhibition is further obstructed by protein C deficiency and by decreased levels of antithrombin, which has its main role of restricting thrombin expression. Moreover, the significant rise in PAI-1 induces the inactivation of fibrinolysis [21]. Just as in DIC, in SIC fibrinolysis suppression is related to the overproduction of PAI-1 and is followed by fibrin clot formation in the tissues’ microcirculation thus leading to organ dysfunction [47]. These conditions can be identified by a decrease in platelet count and an elevation in PT [47].

Even though COVID-19-associated coagulopathy has many similarities to SIC and DIC, it has some notable differences [19], like the fact that in CAC there is an elevation of fibrinogen and D-dimers levels, whereas PT, aPTT and platelet count undergo only small alterations. Other than the biomarkers mentioned, CAC diagnosis also includes the elevation of FVII and VWF levels and an increase in angiopoietin 2—even though its action in CAC is not fully clarified. On the other hand, in SIC the main biomarkers that change enough to be used for diagnosis are PT, platelet count and antithrombin concentration [19].

#### 4.7.2. Venous Thromboembolism (VTE)—Risk Factors, Prognosis, Prophylaxis, Treatment

VTE is a common complication for COVID-19 patients, especially for critically ill ICU patients—despite the thromboprophylaxis [13]. VTE includes DVT (deep vein thrombosis), and PE (pulmonary embolism) and its severity varies depending on the severity of the COVID-19 infection and the patient’s performance status [71]. PE seems to be more common [13], due to thrombus migration and it is not always accompanied by DVT [71]. It is interesting that this high risk of PE in critically ill patients is consistent with pulmonary complications, like pneumonia, during other viral infections, like H1N1 infection [26].

Risk factors for VTE complications in critically COVID-19 ill patients are—other than the common COVID-19 risk factors—higher leukocyte count, higher neutrophil/lymphocyte ratio and elevated D-dimers concentration [12]. It has also been found that higher D-dimers levels, elevated PT, and increased age can be associated with a higher death rate, while a lower death rate is associated with an increase in platelet count [15]. As a matter of fact, in the meta-analysis by Corrêa et al. [17] it was proven that in an ensemble of COVID-19 patients, 23.9% developed VTE, even though they were on anticoagulation therapy. They also indicated that ICU patients had a much higher risk of developing VTE than non-ICU patients, but that does not eliminate the possibility of COVID-19 ward patients coming down with VTE [13]. It should also be mentioned that the lack of thromboprophylaxis in non-ICU patients majorly determined the progress of VTE [13].

A prevention method for VTE, that should be used on all hospitalized COVID-19 patients, is the use of one of the two typical risk assessment tests, which are the PADUA or IMPROVE risk assessment models. Others suggest the use of a screening duplex scan for DVT, done on COVID-19 patients in the ICU every 4–5 days [20]. This test can be guided by clinical suspicion in order to prevent DVT complications [20]. In addition, in patients with higher VTE risk—and low risk of bleeding—thromboprophylaxis is a trustworthy form of prevention and it contains the administration of UFH (unfractioned heparin) or LMWH (low molecular weight heparins), depending on the case [26]. However, since VTE is a common complication in hospitalized COVID-19 patients, it is usually a recommendation that all hospitalized COVID-19 patients without bleeding complication risks, should receive an anticoagulant treatment for VTE prophylaxis [26], in order to prevent thrombotic events and damage in tissues and organs [31].

LMWH and UFH are the drugs of preference—both for VTE prophylaxis and for treatment—over direct oral anticoagulants (DOACs) since the last ones could partake in possible drug-to-drug interaction with antiviral or antibacterial drugs [2]. On the contrary, when it comes to post-hospitalization therapy, DOACs are suggested wince they do not require that much monitoring [61]. Some commonly used LMWH are dalteparin and enoxaparin [72]. LMWH has also been extremely effective in cases of SIC [15] and when patients show a six-fold elevation in D-dimers, thus suggesting LMWH’s usefulness in cases where patients have high D-dimers and SIC symptoms [15].

When it comes to non-ICU hospitalized patients, most guidelines suggest that patients with viral infections such as SARS-CoV-2 infection that are at risk for VTE, should undergo anticoagulant therapy with the use of UFH twice or thrice a day and LMWH once a day [61]. The same medication is also suggested as therapy for VTE hospitalized patients. In every case, the patient-specific VTE risk factors should be taken into consideration before deciding on the type and duration of the therapy [61]. In cases where extreme contradictions are present, mechanical thromboprophylaxis is suggested, even though there is limited evidence of effectiveness in hospitalized patients [61].

As for the VTE prophylaxis for ICU COVID-19 patients, the prognosis is rather poor [61] and it is related to the presence of existing systematic conditions and DIC/SIC symptoms and markers. In these cases, some support that the use of intermediate doses of LMWH in severely ill patients may help with the improvement of the prognosis, but the optimal prophylaxis strategy is uncertain [61].

### 4.8. Coagulation Treatment

#### 4.8.1. Thromboprophylaxis

Thromboprophylaxis is a method that should be used in all hospitalized and immobile patients and not so much in low-risk patients [73]. The main therapy that is suggested is heparin therapy since it is believed to have beneficial effects on COVID-19 patients [73]. Specifically, heparin improves clinical outcomes through its anti-thrombotic, anti-viral and anti-inflammatory activities. However, specific patients that are in the ICU, like obese patients or patients with sepsis, have major thrombotic risks. Thromboprophylaxis with increased doses of LMWH or UFH in ICU patients is in all cases suggested since the thrombotic risk is high [73], except in cases where there are contraindications like hemorrhages [12]. Some data suggest that in specific cases, post-discharge thromboprophylaxis is also needed [13]. For instance, it should be considered for patients with high VTE risk—regardless of SARS-CoV-2 infection—but not for patients with a mild infection that can be treated with at-home quarantine, even if they have other systematic diseases [12].

**Figure 5:**
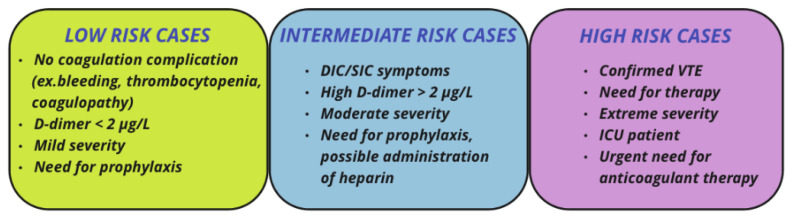
Cases of coagulation complications severity during COVID-19 infections. Every level of severity (low, intermediate, and high) is characterized by different aspects which help both in the diagnosis as well as in the treatment process.

Thromboprophylaxis is rather important since it can determine the progress of the disease and control possible complications. Therefore, it should be closely monitored. The medication plan depends both on the extent of the complications as well as on the patient-specific parameters [15] (Figure 5).

#### 4.8.2. Treatment for Endothelial Damage

Therapy for endothelial damage includes synthetic serine protease inhibitors like nafamostat mesylate and camostat mesylate, which tend to prevent SARS-CoV-2 infection by inhibiting TMPRSS2 at the stage of viral entry in the host cells [48]. Other than that, nafamostat mesylate also has anticoagulatory effects, and in some cases in Japan, it has been used to treat DIC [48]. This part of therapy also contains physiologic anticoagulants, such as protein C and antithrombin. Specifically, protein C, when triggered, inactivates FVIIIa and upregulates ACE2, while quelling coagulation and inflammation. By suppressing the inflammatory response, protein C also protects from pulmonary injury. At the same time, antithrombin is a serine protease that not only restricts many coagulation factors but also protects the glycocalyx by binding to heparan sulfate [48].

#### 4.8.3. Heparin

The use of heparin and heparin-based products (LMWH, UFH) as an anticoagulant is quite common. When it comes to heparin therapy, its monitoring during inflammation can be difficult, especially when using UFH [22]. This is a result of the fact that in cases of serious coagulopathy, the use of aPTT for observing heparin treatment with UFH can be obstructed by the fact that aPTT cannot be used in acute-phase conditions, since it can either be prolonged or shortened and subsequently affect the results of the anticoagulant effect of UFH [22]. For this reason, some suggest that anti-Xa levels should be monitored during UFH and LMWH therapy, instead of aPTT, as prolonged aPTT with elevated levels of FVIII and positive LAs is common [19,74]. Heparin administration sometimes can result in heparin-induced thrombocytopenia (HIT), which is a drug reaction that results in a prothrombotic complication [13].

Other than their anticoagulant activity, heparins also have anti-inflammatory activity, as they induce reductions in IL-6, IL-8 and in pulmonary microvascular endothelial damage [26]. Furthermore, heparin blocks neutrophil chemotaxis and eosinophil migration, as well as the adhesion of leukocytes to the endothelium, which is an early sign of sepsis [75]. It has been demonstrated that heparin products, like LMWH and UFH, manage not only to unbalance the domain of SARS-CoV-2 that binds to the receptor but also to spot the protein’s binding to the ACE2 receptor [26].

#### 4.8.4. Iron Chelation

Iron deficiency is a quite common condition and can lead to many problems, with one of them being the impairment of host immunity [8]. At the same time, iron overload can create oxidative stress which results in viral mutations [8]. During COVID-19, ferritin levels are elevated and cause detrimental effects on the endothelium and on the coagulation mechanisms. Iron chelation is a therapeutic approach that is believed to attack viral infection and is attainable through the administration of Deferoxamine or Deferiprone [8]. Bearing in mind the effect of iron on the virus and the results of previous studies, it is believed that inhibition of iron supply to the virus can help in treatment, as long as characteristics, like serum iron and ferritin levels are known and understood [8].

#### 4.8.5. Convalescent Plasma Therapy

Convalescent plasma therapy (CPT) has been previously used to treat viral infections like H1N1 and Ebola. During this process, the plasma transfusion leads to passive immunization, which inhibits viremia and improves the progress of the infection [8]. Some believe that CPT brings no significant improvement in COVID-19 patients [76], while another analysis showed that CPT could be beneficial in advanced cases [77]. This was supported by a meta-analysis that proved that CPT leads to a decrease in CRP levels [78]. Even though CPT is suggested in limited cases, it is believed that can be beneficial for COVID-19 patients, especially during the first week of the infection, when viremia spikes [79]. Generally, this practice is not commonly carried out.

#### 4.8.6. Antiplatelets—Aspirin, P2Y12 Receptor Antagonist, Dipyridamole

Acetylsalicylic acid or commonly known as aspirin is mainly studied in ARDS [23]. Both animal and human studies focused on aspirin showed that it can have beneficial effects on ARDS and improve survival chances in cases of severe lung injury. It is believed to reduce mortality both for ICU patients and during prehospital use [80]. However, the use of aspirin during COVID-19 infection has yet to be brought to light [23].

When it comes to P2Y12 Receptor Antagonists, there is not much evidence of their COVID-19 use, however, its administration in cases of pneumonia can lead to a decrease in circulating platelet and leukocyte count, lower IL-6 levels and improved lung function and oxygen requirements [23].

Dipyridamole (DIP) has an antithrombotic effect and the role of an antiplatelet agent that acts as a phosphodiesterase (PDE) inhibitor which increases intracellular cAMP/cGMP [14,23]. DIP other than its common efficacies has recently been demonstrated as a therapeutic factor for COVID-19 by suppressing SARS-CoV-2 replication in vitro [14], while also increasing lymphocyte and platelet count and decreasing D-dimers concentration [14]. However, we need well-designed studies to ensure DIP’s role in the treatment of COVID-19.

#### 4.8.7. Anti-inflammatory Agents—Corticosteroids, Hydroxychloroquine, Statins

Corticosteroids may regulate thrombotic risk through anti-inflammatory activity [23]. It is possible that glucocorticoids can regulate inflammation and coagulation factors like fibrinogen and vWF. COVID-19 treatment with these in patients with ARDS complication suggested a reduced risk of death [23]. However, experiments have shown that steroids are also linked to increased levels of clotting molecules and exogenous glucocorticoids are associated with thrombotic risk [81,82,83], however further research is needed.

Even though hydroxychloroquine shows no evidence regarding COVID-19 thrombogenesis, it is known to have antithrombotic activity in patients with diseases like lupus erythematosus, rheumatoid arthritis, and antiphospholipid syndrome [23]. Studies suggest that antiphospholipid antibodies have antiplatelet effects and reversal thrombogenic properties [23].

As for statins, they have anti-inflammatory, as well as antiplatelet and anticoagulant effects. Moreover, prior studies have proven that they can reduce rates of VTE as well as stabilize atherosclerotic plaques [84]. Therefore, they can be useful in COVID-19 cases, where thrombotic and inflammatory complications occur [85].

#### 4.8.8. Targeted Immunomodulatory Therapies

JAK inhibitors and complement cascade inhibition in general could be potential therapeutic approaches for COVID-19. A drug that can be used to block the complement cascade is eculizumab, which is an anti-C5 monoclonal antibody that inhibits terminal complement activation. Some drugs that act as JAK inhibitors and could potentially be used against SARS-CoV-2 are baricitinib, ruxolitinib and tocilizumab. Baricitinib, which is used for treating rheumatoid arthritis, has in vitro activity against COVID-19. However, it has contraindications because it is associated with risk for VTE development. Similarly, ruxolitinib is used for myelofibrosis and polycythemia vera but could also be used against SARS-CoV-2 [23].

#### 4.8.9. Activated Protein C

Activated protein C (APC) has antithrombotic effects in early sepsis-induced DIC and may restrict the detrimental effects caused by ischemia or sepsis. Moreover, APC has anti-inflammatory effects through the pathway that is mediated by PAR1 [23,86]. The use of APC or its mutants in COVID-19 patients, even those with DIC, has potential and should be further investigated [23].

#### 4.8.10. Hemostatic Modulating Agents—Antithrombin, Contact Activation System

Antithrombin levels of COVID-19 are much lower than the levels of healthy individuals [87]. It is possible that the administration of antithrombin can modulate inflammation and coagulopathy [23]. However, there is still no clear connection between the use of antithrombin and critically ill COVID-19 patients.

The contact activation system includes FXII, FXI, high molecular weight kininogen (HK) and prekallikrein (PK) and it links inflammation and coagulation by inducing the production of thrombin and bradykinin. As already discussed, thrombin promotes clot formation and platelet activation while bradykinin helps with the release of proinflammatory cytokines. Some nonhuman models suggest that inhibition of the contact activation system can lower inflammatory cytokine levels, microvascular thrombosis and contribute to survival [23].

## 5. Conclusions

It is obvious that complications in COVID-19 hospitalized patients are common and highly affect the progress of the infection. However, there are still many gaps when it comes to the prognosis, diagnosis and treatment of cases as such. In other words, the prognosis of hospitalized critically ill COVID-19 patients is still poor and SARS-CoV-2 infection is still an unpredictable challenge. A vital step forward is the development of effective and safe protocols for thromboprophylaxis and thrombosis treatment during COVID-19 disease. Bearing this in mind, it is necessary that the role of all biomarkers is completely clarified and an optimal protocol for the management of every case is established.

Summing up, coagulopathy and hemostatic imbalance are frequent conditions during COVID-19 hospitalization. To prevent further complications, it is important to aim for early diagnosis and to complete a series of tests, which can determine the prognosis and risk level of each patient. Based on that, early thromboprophylaxis should be administered, and in more profound and advanced cases, the right antithrombotic or anti-inflammatory treatment strategy should be implemented.

## Data Availability

Not applicable.
